# Identification and optimization of the key growth parameters involved in carotenoids production of the marine microalga *Pavlova gyrans*

**DOI:** 10.1038/s41598-024-66986-y

**Published:** 2024-07-26

**Authors:** Filipe Maciel, Paulo Berni, Pedro Geada, José Teixeira, Joana Silva, António Vicente

**Affiliations:** 1 CEB - Centre of Biological Engineering, University of Minho, Campus de Gualtar, Braga, Portugal; 2 LABBELS –Associate Laboratory, Braga, Guimarães, Portugal; 3 ALLMICROALGAE, Natural Products S.A., Lisbon, Portugal

**Keywords:** Applied microbiology, Industrial microbiology

## Abstract

In this work, a multivariate analysis was carried out, using a Plackett–Burman (PB) design involving seventeen growth parameters, on carotenoids production of *Pavlova gyrans* (*p* < 0.10). Each assay was analysed regarding its content (mg g^−1^) of fucoxanthin (Fx), diatoxanthin, diadinoxanthin, β-carotene (βCar), α-carotene, and the sum of all carotenoids analysed individually (TCar). According to the statistical analysis, modified medium formulations were developed for the particular cases of Fx, βCar, and TCar. The study showed that Fx content was positively affected by nitrogen supplementation and lower light intensities. Higher concentrations of nitrogen and iron increased the final content of βCar as well. Similarly, salinity, light intensity, nitrogen, iron, and cobalt were identified as key factors in TCar production. The PB-based formulations showed significant improvements (*p* < 0.05) for TCar (11.794 mg g^−1^) and Fx (6.153 mg g^−1^) when compared to the control conditions (Walne’s medium—2.010 mg g^−1^). Furthermore, effective control of key variables (e.g., light intensity) throughout *P. gyrans* growth proved successful (*p* < 0.05), increasing the productivity of Fx (0.759 mg L^−1^ d^−1^) and TCar (1.615 mg L^−1^ d^−1^).

## Introduction

Carotenoids are the pigments responsible for the yellow, orange, and red colors in nature, such those presented by microalgae, bacteria, and plants^1^. They have been highlighted as high added-value products due to their biological activity and coloring properties, which make them of great interest for biotechnological and industrial applications^2^. Most of these functions are associated with the antioxidant properties of carotenoids due to their ability to quench singlet oxygen and reactive oxygen species, which is strongly dependent of the pigments’ chemical composition^3,4^.

Humans are not able to naturally synthesize carotenoids and their needs are only met through proper nutrition. Thus, considering their biological relevance along with the consumers’ awareness for a healthy lifestyle, there has been an increasing demand for carotenoids in recent decades, estimating a compound annual growth rate of 5.7% and a potential global market value of 2.7 billion US$ by 2027^5^. Cheaper production of chemically synthetized colorants makes them attractive, however, they represent a high environmental impact and health concerns raised when used as ingredient for human consumption^6^. Furthermore, synthetic carotenoids are not a viable option since they have significantly lower bioactivity compared to the natural ones^7^.

Microalgae have been presented as one of the most promising alternative sources of natural carotenoids. These microorganisms are widespread in nature and are known for their high photosynthetic activity, fast growth, and metabolic plasticity. They possess a rich composition, which can include several bioactive pigments, polyunsaturated fatty acids and/or high protein content^8–10^. Several species have been commercially explored as producers of carotenoids, being the most well-known examples *Haematococcus pluvialis* and *Dunaliella salina*, which can produce 6% and 10% of their dry weight (DW) as astaxanthin and βCar, respectively^11,12^.

Nevertheless, the differentiated properties of natural carotenoids, along with challenging implementation of microalgae production at industrial scale, contributed to their increasing market value, which can reach 1500 US$ kg^−1^ for βCar or even 42,000 US$ kg^−1^ for Fx^13^. Thus, to improve the cost-effectiveness of the process, several research studies have been carried out with the aim of identifying microalgae species enriched in carotenoids and evaluating growth conditions and strategies to improve biomass production and carotenoids accumulation^14,15^.

Recently, microalgae from the Pavlovophyeceae class have been seen as a promising source of bioactive compounds for industries other than aquaculture, such as food and pharmaceutical industries or even wastewater management^16–19^. Firstly, its commercial interest relied on their ability to produce large lipids contents enriched with the high valuable eicosapentaenoic acid (EPA) and docosahexaenoic acid (DHA)^20^. However, these microalgae also present an interesting composition of sterols, essential amino acids, higher digestibility due to the absence of cell wall and a valuable carotenoids profile composed of diadinoxanthin (Ddx), diatoxanthin (Dtx), βCar and, mostly, Fx (51–68% of total carotenoids)^18,20,21^. As previously mentioned, carotenoids have been highlighted and highly demanded due to their biological properties. Among them, Fx has shown enormous potential and interest for the nutraceutical market given its differentiated and high bioactivity, such as anti-obesity^22^, anti-cancer^23^, and anti-diabetic effect^24^.

The present study aims to optimize the carotenoids accumulation of the microalga *Pavlova gyrans* through a multivariate approach involving seventeen growth parameters. As described in Maciel, F. et al^25^, three abiotic factors stood out among seventeen as the most significant for the growth of *P. gyrans*: light intensity, NaNO_3_ and CuSO_4_.5H_2_O. These three variables, together with phosphorus, were optimised, showing a 3.8-fold increase in biomass production. The optimal combination obtained validates the positive effect of greater irradiation and nitrogen and phosphorus supplementation, while copper promoted superior growth when supplied in lower concentrations^25^. To the authors' knowledge, and unlike other haptophytes (*e.g. Tisochrysis lutea*) and diatoms (*e.g. Phaeodactylum tricornutum*), few studies were found that include the simultaneous study of a large number of growth conditions on carotenoids content—and, in particular, on Fx—of Pavlovophyeceae species, despite their potential as carotenoids source. Throughout the optimization process, several growth conditions were formulated and validated against the control conditions. Moreover, the most significant growth parameters were also validated against the optimum growth conditions for biomass production of *P. gyrans*, which were determined in a previous work of our research group^25^.

## Material and methods

### Microalga and inoculum preparation

The non-axenic microalga strain *Pavlova gyrans* (RCC1553) was obtained from the Roscoff Culture Collection (Roscoff, France). It was maintained in Walne’s medium (500 mg L^−1^ NaNO_3_), at Level 0 (Table 1), under salinity of 30 psu^26^. The inoculum was up-scaled to 2 L flat bottom flask, in which was bubbled with a mixture of air and CO_2_ (8 mL min^−1^—Alicat Scientific, USA) to keep the pH value in the range of 8.0 ± 0.5. The cultures were grown at room temperature (22 to 26 °C) and laterally irradiated with white light emitting diodes (LEDs—100 µmol photons m^−2 ^s^−1^) over 24 h. Photosynthetically active radiation (PAR) was measured with a Li-250A light meter equipped with Q44069 sensor.

**Table 1:** The seventeen abiotic factors, and respective levels, assessed in the carotenoids composition of *P. gyrans* through the Plackett–Burman experimental design.

Abiotic factor		Level		
		− 1	0	1
*x*_*1*_	Inoculum size (AFDW g L^−1^)	0.1	0.2	0.3
*x*_*2*_	Salinity (psu)	20	30	40
*x*_*3*_	Light intensity (µmol photons m^−2^ s^−1^)	150	450	750
*x*_*4*_	Air flow (mL min^−1^)	600	800	1000
*x*_*5*_	NaNO_3_ (mg L^−1^)	250	500	750
*x*_*6*_	NaH_2_PO_4_ H_2_O (mg L^−1^)	10	20	30
*x*_*7*_	Na_2_H_2_EDTA 2H_2_O (mg L^−1^)	22.5	45	67.5
*x*_*8*_	H_3_BO_3_ (mg L^−1^)	16.8	33.6	50.4
*x*_*9*_	FeCl_3_ 6H_2_O (mg L^−1^)	0.65	1.3	1.95
*x*_*10*_	MnCl_2_ 4H_2_O (µg L^−1^)	180	360	540
*x*_*11*_	NaHCO_3_ (mg L^−1^)	170	652	1134
*x*_*12*_	ZnCl_2_ (µg L^−1^)	10.25	21	31.75
*x*_*13*_	CoCl_2_ 6H_2_O (µg L-^1^)	10	20	30
*x*_*14*_	(NH_4_)_6_Mo_7_O_24_ 4H_2_O (µg L^−1^)	4.5	9	13.5
*x*_*15*_	CuSO_4_ 5H_2_O (µg L^−1^)	10	20	30
*x*_*16*_	Thiamin (µg L^−1^)	50	100	150
*x*_*17*_	Cyanocobalamin (µg L^−1^)	2.5	5	7.5

### Screening of the growth parameters for carotenoids production by *P. gyrans* using a Plackett–Burman design

The experimental design adopted to assess the impact of growth factors on the carotenoids composition of *P. gyrans* comprise the experiments performed in Maciel, F. et al*.*^25^, Table 1. Each factor was evaluated at three different levels: − 1, 0, and 1. The corresponding combinations of the growth conditions are presented in Table 2. Briefly, seventeen growth parameters were assessed through a Plackett–Burman design (PB), composed of 24 individual combinations and 4 central points (assays 25–28), totaling 28 assays^27^. All independent variables were set at Level 0 in the central points, which allowed evaluating the repeatability of the carotenoids’ composition of *P. gyrans*. The experiments were carried out randomly in three separated experimental runs, using 1 L bubble column reactors (glass, 6.5 cm diameter and 43 cm high—see supplementary material) at room temperature and laterally illuminated with LEDs lighting. Salinity was adjusted using a concentrated sea salt stock solution and measured with a seawater refractometer (Hanna HI 96,822, USA). Reactors were continuously aerated with filtered air (0.2 µm) through an internal glass tube located at the center of the reactor. CO_2_ was supplied in-line (0.6 mL min^−1^) to keep pH = 8.0 ± 0.5 (Hanna HI 2210, USA).

**Table 2:** Coded values of each independent variable (*x*) used in the twenty-eight assays performed in the Plackett–Burman design.

*x*	Assay																											
	1	2	3	4	5	6	7	8	9	10	11	12	13	14	15	16	17	18	19	20	21	22	23	24	25	26	27	28
1	1	1	1	1	1	− 1	1	− 1	1	1	− 1	− 1	1	1	− 1	− 1	1	− 1	1	− 1	− 1	− 1	− 1	− 1	0	0	0	0
2	− 1	1	1	1	1	1	− 1	1	− 1	1	1	− 1	− 1	1	1	− 1	− 1	1	− 1	1	− 1	− 1	− 1	− 1	0	0	0	0
3	− 1	− 1	1	1	1	1	1	− 1	1	− 1	1	1	− 1	− 1	1	1	− 1	− 1	1	− 1	1	− 1	− 1	− 1	0	0	0	0
4	− 1	− 1	− 1	1	1	1	1	1	− 1	1	− 1	1	1	− 1	− 1	1	1	− 1	− 1	1	− 1	1	− 1	− 1	0	0	0	0
5	− 1	− 1	− 1	− 1	1	1	1	1	1	− 1	1	− 1	1	1	− 1	− 1	1	1	− 1	− 1	1	− 1	1	− 1	0	0	0	0
6	1	− 1	− 1	− 1	− 1	1	1	1	1	1	− 1	1	− 1	1	1	− 1	− 1	1	1	− 1	− 1	1	− 1	− 1	0	0	0	0
7	− 1	1	− 1	− 1	− 1	− 1	1	1	1	1	1	− 1	1	− 1	1	1	− 1	− 1	1	1	− 1	− 1	1	− 1	0	0	0	0
8	1	− 1	1	− 1	− 1	− 1	− 1	1	1	1	1	1	− 1	1	− 1	1	1	− 1	− 1	1	1	− 1	− 1	− 1	0	0	0	0
9	− 1	1	− 1	1	− 1	− 1	− 1	− 1	1	1	1	1	1	− 1	1	− 1	1	1	− 1	− 1	1	1	− 1	− 1	0	0	0	0
10	− 1	− 1	1	− 1	1	− 1	− 1	− 1	− 1	1	1	1	1	1	− 1	1	− 1	1	1	− 1	− 1	1	1	− 1	0	0	0	0
11	1	− 1	− 1	1	− 1	1	− 1	− 1	− 1	− 1	1	1	1	1	1	− 1	1	− 1	1	1	− 1	− 1	1	− 1	0	0	0	0
12	1	1	− 1	− 1	1	− 1	1	− 1	− 1	− 1	− 1	1	1	1	1	1	− 1	1	− 1	1	1	− 1	− 1	− 1	0	0	0	0
13	− 1	1	1	− 1	− 1	1	− 1	1	− 1	− 1	− 1	− 1	1	1	1	1	1	− 1	1	− 1	1	1	− 1	− 1	0	0	0	0
14	− 1	− 1	1	1	− 1	− 1	1	− 1	1	− 1	− 1	− 1	− 1	1	1	1	1	1	− 1	1	− 1	1	1	− 1	0	0	0	0
15	1	− 1	− 1	1	1	− 1	− 1	1	− 1	1	− 1	− 1	− 1	− 1	1	1	1	1	1	− 1	1	− 1	1	− 1	0	0	0	0
16	1	1	− 1	− 1	1	1	− 1	− 1	1	− 1	1	− 1	− 1	− 1	− 1	1	1	1	1	1	− 1	1	− 1	− 1	0	0	0	0
17	− 1	1	1	− 1	− 1	1	1	− 1	− 1	1	− 1	1	− 1	− 1	− 1	− 1	1	1	1	1	1	− 1	1	− 1	0	0	0	0

Microalgal growth was monitored by optical density (750 nm) every 2 days, being this value subsequently converted to ash-free dry weight (AFDW) using the following calibration curve: $AFDW({\text{g}}\;{\text{L}}^{ - 1} {)} = {0}{\text{.8991}} \times OD_{750} { + 0}{\text{.0054,}}$ *R*^2^ $=$ 0.99. The experiments were stopped once the stationary growth phase was attained and the cultures were centrifuged at 2147 *g* for 20 min (Centurion Pro-Analytical CR7000, Chichester, United Kingdom). The pellets were recovered and stored at – 20 °C, and further at − 80 °C for lyophilization.

#### Validation test

The calculated effects and corresponding statistical significance of the PB design enabled the identification of the most relevant growth conditions for each carotenoid assessed and the sum of all carotenoids (mg g^−1^) in *P. gyrans*. That information was used to define tailored growth conditions for the carotenoid of interest, in which the carotenoids’ yields were evaluated and validated in two new different sets of experiments (V1 and V2). The full composition of the growth conditions used in both sets of experiments is described in Table 3. The first set (V1) aimed at validating the optimum conditions defined in PB design for the dependent variables Fx, TCar, and βCar, against the control conditions (Con—Walne’s medium). For practical reasons, the variables inoculum size (*x*_*1*_), light intensity (*x*_*3*_), and air flow (*x*_*4*_) in the control assay were the same of the remaining experiments. The non-significant growth parameters factors (*p* > 0.10) for Fx, βCar and TCar were set at Level -1.

**Table 3:** Levels of the growth parameters used in the validation experiments.

	V1				V2			
	Con	Fx	βCar	TCar	Opt	Fx1ph	Fx2ph	TCar
Inoculum size (AFDW g L^−1^)	0.1	0.1	0.1	0.1	0.1	0.1	0.1	0.1
Salinity (psu)	30	20	20	**20**	30	30	30	**20**
Light intensity	150	**150**	150	**150**	700	**150**	**700/150**	**150**
Air flow (mL min^−1^)	600	600	600	600	600	600	600	600
NaNO_3_ (mg L^−1^)	100	**750**	**750**	**750**	1500	**1500**	**1500**	**1500**
NaH_2_PO_4_ H_2_O (mg L^−1^)	20	10	10	10	40	40	40	40
Na_2_H_2_EDTA 2H_2_O (mg L^−1^)	45	22.5	22.5	22.5	45	45	45	45
H_3_BO_3_ (mg L^−1^)	33.6	16.8	16.8	16.8	33.6	33.6	33.6	33.6
FeCl_3_ 6H_2_O (mg L^−1^)	1.3	0.65	**1.95**	**1.95**	1.3	1.3	1.3	**1.95**
MnCl_2_ 4H_2_O (µg L^−1^)	360	180	180	180	360	360	360	360
NaHCO_3_ (mg L^−1^)	–	170	170	170	170	170	170	170
ZnCl_2_ (µg L^−1^)	21	10.25	10.25	10.25	21	21	21	21
CoCl_2_ 6H_2_O (µg L^−1^)	20	10	10	**30**	20	20	20	**30**
(NH_4_)6Mo_7_O_24_ 4H_2_O (µg L^−1^)	9	4.5	4.5	4.5	9	9	9	9
CuSO_4_ 5H_2_O (µg L^−1^)	20	10	10	10	6	6	6	6
Thiamin (µg L^−1^)	100	50	50	50	100	100	100	100
Cyanocobalamin (µg L^−1^)	5	2.5	2.5	2.5	5	5	5	5

In V1, Walne's medium (control—Con) was compared to the maximized conditions for accumulation of fucoxanthin (Fx), β-carotene (βCar), and the sum of all carotenoids analyzed (TCar). The set V2 represents the maximized conditions for TCar and fucoxanthin accumulation using the optimized growth conditions for *P. gyrans’* biomass production (Opt). Two strategies for fucoxanthin production were evaluated: 150 µmol photons m^−2^ s^−1^ during the entire growth (Fx1ph) and a two-phase growth (Fx2ph) using 700 µmol photons m^−2 ^s^−1^ for the first 8 days and 150 µmol photons m^−2^ s^−1^ for the last 2 days (stationary phase). Bold numbers represent the most significant variables and their values according to the calculated effects in Plackett–Burman. Underlined numbers represent the optimum growth conditions achieved for the maximal biomass production of *P. gyrans.*

The second set of validation experiments (V2) was devised to understand if the variables identified as being the most significant in the PB design (*p* < 0.10) could trigger the accumulation of carotenoids in *P. gyrans* when cultured under the optimum growth conditions (Opt) for biomass production, as described in a previous work of our research group^25^. Briefly, Opt (Table 3) presents the optimal values for the growth factors: light intensity (*x*_*3*_), NaNO_3_ (*x*_*5*_), NaH_2_PO_4_ H_2_0 (*x*_*6*_), and CuSO_4_ 5H_2_O (*x*_*15*_). Regarding the non-significant independent variables identified in the PB matrix for biomass production, inoculum size (*x*_*1*_), airflow (*x*_*4*_), and NaHCO_3_ concentration (*x*_*11*_) were set at Level -1, while the remaining growth parameters were set at Level 0. In addition to the assay Opt, that was used as control, this formulation was modified to promote the accumulation of all carotenoids (TCar), and fucoxanthin (Fx1ph and Fx2ph) Table 3.

Regarding Fx, two different strategies were applied. In the assay Fx1ph, the whole growth of *P. gyrans* was performed under the same light intensity (150 µmol photons m^−2^ s^−1^). On the other hand, and according to the light intensity used, the growth in the Fx2ph experiment was divided into two phases. First, *P. gyrans* was grown at the optimal light intensity for biomass production (700 µmol photons m^−2^ s^−1^) until the end of the exponential growth phase; in the second phase, lasting 2 days, the light intensity was reduced to 150 µmol photons m^−2^ s^−1^ to increase the Fx content of *P. gyrans*. Since NaNO_3_ showed a positive effect on Fx and TCar, and the concentration defined in the maximized growth conditions for biomass production was higher than those set in the PB design, the V2 experiments were supplemented with 1500 mg L^−1^ of NaNO_3_. All experiments were performed in triplicate.

### Biomass characterization

#### Pigment extraction, identification, and quantification by HPLC–DAD

Pigment analysis was conducted as described by Sanz et al.^28^ with some modifications. Carotenoids were extracted from 10 mg of freeze-dried biomass in a screw cap glass tube with 3 mL of cold extraction solution (90% acetone, 0.1% BHT). Tubes were kept in ice and protected from light during all steps. Samples were left extracting for 15 min to allow acetone penetrating in microalgae structures, then they were vortexed for 30 s followed by 5 min in an ultrasonic bath. Extracts were centrifuged for 15 min at 358 *g* (Hettich Mikro 120, Tuttlingen, Germany) and the supernatant was collected. The pellet was re-extracted until it was colorless. From the total extract, 1 mL was syringe filtered (0.22 µm nylon) to an amber vial, and received 0.3 mL of ultra-pure H_2_O to avoid incompatibility of solvents in the HPLC–DAD^29^.

The extracts were analyzed in a Shimadzu Nexera X2 system equipped with a 30AD liquid chromatograph, a SIL-30AC autosampler, a CTO-20AC column oven, a SPD-M20A diode array detector, and a CBM-20A Communication Bus Module. The pigments separation was performed through an ACE C18 PFP column 150 mm × 4.6 mm, 3 µm particle size (Advanced Chromatography Technologies, Aberdeen, Scotland) at 40 °C. The mobile phase was a mixture of methanol:225 mM ammonium acetate (82:18, v:v) as eluent A and ethanol as eluent B. The gradient followed the indications of Sanz et al.^28^ with some modifications: 96%:4% eluent A:eluent B (0–16 min), 62%:38% eluent A:eluent B (16–22 min), 62%:38% eluent A:eluent B (22–28 min), 28%:72% eluent A:eluent B (28–35 min), 20%:80% eluent A:eluent B (35–45 min), 20%:80% eluent A:eluent B (45–50 min) and 96%:4% eluent A:eluent B (50–55 min). The flow rate was 1 mL min^−1^, the run duration 55 min, and the injection volume 20 µL. All reagents used in pigment extraction and chromatography analysis were HPLC grade. Identification of the extracted pigments was accomplished by comparison of the retention times and absorption spectra with commercial standards. Standards for chlorophyll *a*, chlorophyll *c*_*1*_, chlorophyll *c*_*2*_, fucoxanthin, all-*trans*-β-carotene, diadinoxanthin, and diatoxanthin were obtained from DHI (Hørsholm, Denmark). The identification of α-carotene (αCar) was done only by absorption spectra and retention time. All analysis were performed in triplicate.

#### Biomass and carotenoids productivity of *P. gyrans*

Volumetric productivity of the carotenoids (*P*_*C*_) and the biomass (*P*_*X*_) produced by *P. gyrans* was calculated using the following equations:

$$P_{C} \left( {{\text{mg}}\;{\text{L}}^{ - 1} \;{\text{d}}^{ - 1} } \right) = \frac{{C_{i} \times \left( {X_{tf} - X_{t0} } \right)}}{{t_{f} }}$$

$$P_{X} \left( {{\text{g}}\;{\text{L}}^{ - 1} \;{\text{d}}^{ - 1} } \right) = \frac{{X_{tf} - X_{0} }}{{t_{t} }}$$

where *C*_*i*_ is the carotenoid content, mg g^−1^, and *X* is the ash-free dry weight, g L^−1^, of the sampling time, days, at the end (*t*_*f*_) and the beginning (*t*_*0*_) of *P. gyrans* growth.

### Statistical analysis

The growth parameters defined in the PB design as the most significant in the carotenoids production of *P. gyrans* were identified using a 90% confidence level (*p* < 0.10), in order to avoid excluding any important independent variable^27^. The statistical analysis was performed with the online software Protimiza Experimental Design (http://experimental-design.protimiza.com.br/). The validation tests were evaluated for differences between means using one-way ANOVA followed by Tukey’s multiple comparison test at 95% confidence level (GraphPad Prism version 8.0.2).

## Results and discussion

### Plackett–Burman design

Regarding the pigments composition of *P. gyrans*, the chromatogram achieved during the preliminary tests (see supplementary material), overall, allowed to confirm the pigment profile commonly described for this species and other Pavlovophyceae species^21,30^. Eight pigments were identified: three chlorophylls and five carotenoids. Despite the identification of chlorophylls *a*, *c*_*1*_ and* c*_*2*_, they were not quantified because the goal of this work was to study the ability of *P. gyrans* to accumulate carotenoid pigments, reason why statistics and discussion are focused on carotenoids. Among the carotenoids, it was possible to identify fucoxanthin (Fx), diadinoxanthin (Ddx), diatoxanthin (Dtx), β-carotene (βCar) and α-carotene (αCar), which match to the carotenoid composition described in other strains of *P. gyrans*^30^.

According to their role on microalgae, these pigments can be classified as primary pigments—if they are part of the light-harvesting complexes (LHCs), located in thylakoid membranes, and are involved in light capture and photosynthetic activity^31^—or secondary pigments—those with a photoprotective role and which production is triggered under stressful growth conditions. Among the pigments present in *P. gyrans,* chlorophylls* a*,* c*_*1*_, and *c*_*2*_, as well as Fx, are part of the LHCs, while Ddx, Dtx, and βCar act as a photoprotective agents^32^.

The influence of the twenty-eight growth parameters combinations tested on the carotenoids’ composition of *P. gyrans* is present in Table 4. Fx content ranged from 3.409 mg g^−1^ DW in assay #13 to 0.401 mg g^−1^ DW in assay #3. The extreme values for Ddx were obtained in the assays #13 and #3 with 1.068 and 0.113 mg g^−1^ DW, respectively, whereas the Dtx content varied from 0.091 (#4) to 1.678 mg g^−1^ DW (#17). Regarding the carotene production, the highest values of βCar and αCar were 0.491 (#11) and 0.545 mg g^−1^ DW (#9) while the lowest were 0.151 (#27) and 0.114 mg g^−1^ DW (#18). Finally, and summing all the carotenoids previously mentioned, the total carotenoids content ranged from 6.743 (#13) to 1.017 mg g^−1^ DW (#4). The wide variation achieved for each carotenoid, as well as its total content, represents well the key role of the growth conditions and nutrient availability in microalgae metabolism, which in this case had as main outcome a diversified carotenoids’ composition.

**Table 4:** Carotenoids composition, mg g^−1^, of *P. gyrans* produced in the Plackett–Burman design.

#Assay	Fucoxanthin (mg g^−1^ DW)	Diatoxanthin (mg g^−1^ DW)	Diadinoxanthin (mg g^−1^ DW)	β-carotene (mg g^−1^ DW)	α-carotene (mg g^−1^ DW)	Total carotenoids (mg g^−1^ DW)
1	1.670 ± 0.047	0.707 ± 0.014	0.427 ± 0.005	0.342 ± 0.005	0.413 ± 0.008	3.587 ± 0.052
2	1.942 ± 0.008	0.620 ± 0.002	0.341 ± 0.003	0.257 ± 0.002	0.265 ± 0.002	3.426 ± 0.018
3	0.401 ± 0.002	0.093 ± 0.000	0.113 ± 0.000	0.277 ± 0.001	0.242 ± 0.060	1.127 ± 0.003
4	0.444 ± 0.000	0.091 ± 0.000	0.113 ± 0.000	0.231 ± 0.002	0.137 ± 0.002	1.017 ± 0.004
5	0.933 ± 0.007	0.183 ± 0.002	0.184 ± 0.001	0.387 ± 0.001	0.421 ± 0.001	2.107 ± 0.011
6	1.417 ± 0.024	0.190 ± 0.001	0.282 ± 0.014	0.285 ± 0.015	0.342 ± 0.019	2.517 ± 0.023
7	0.570 ± 0.008	0.200 ± 0.001	0.194 ± 0.001	0.278 ± 0.013	0.193 ± 0.008	1.437 ± 0.023
8	2.161 ± 0.017	0.334 ± 0.003	0.554 ± 0.007	0.174 ± 0.009	0.245 ± 0.011	3.468 ± 0.045
9	0.765 ± 0.002	0.257 ± 0.001	0.410 ± 0.003	0.482 ± 0.003	0.545 ± 0.004	2.460 ± 0.013
10	1.118 ± 0.011	0.331 ± 0.006	0.207 ± 0.006	0.289 ± 0.007	0.338 ± 0.008	2.282 ± 0.035
11	0.846 ± 0.013	0.184 ± 0.003	0.167 ± 0.051	0.491 ± 0.009	0.461 ± 0.005	2.149 ± 0.027
12	0.482 ± 0.002	0.323 ± 0.001	0.247 ± 0.001	0.225 ± 0.008	0.194 ± 0.008	1.471 ± 0.016
13	3.409 ± 0.068	1.474 ± 0.017	1.068 ± 0.005	0.396 ± 0.002	0.395 ± 0.003	6.743 ± 0.078
14	1.896 ± 0.035	0.798 ± 0.009	0.393 ± 0.015	0.210 ± 0.007	0.221 ± 0.008	3.518 ± 0.022
15	0.469 ± 0.004	0.258 ± 0.003	0.183 ± 0.001	0.244 ± 0.004	0.230 ± 0.004	1.385 ± 0.014
16	0.421 ± 0.10	0.415 ± 0.011	0.237 ± 0.007	0.191 ± 0.002	0.191 ± 0.002	1.454 ± 0.023
17	3.290 ± 0.015	1.678 ± 0.008	0.991 ± 0.007	0.312 ± 0.003	0.286 ± 0.003	6.557 ± 0.036
18	3.351 ± 0.024	0.476 ± 0.003	0.767 ± 0.005	0.206 ± 0.003	0.114 ± 0.001	4.915 ± 0.036
19	0.467 ± 0.033	0.134 ± 0.010	0.154 ± 0.009	0.250 ± 0.020	0.173 ± 0.014	1.178 ± 0.086
20	1.850 ± 0.024	0.488 ± 0.007	0.375 ± 0.006	0.195 ± 0.005	0.213 ± 0.007	3.121 ± 0.044
21	0.799 ± 0.006	0.242 ± 0.002	0.322 ± 0.002	0.463 ± 0.004	0.642 ± 0.005	2.468 ± 0.013
22	1.651 ± 0.167	0.401 ± 0.049	0.600 ± 0.074	0.285 ± 0.046	0.352 ± 0.056	3.290 ± 0.363
23	1.599 ± 0.213	0.836 ± 0.127	0.526 ± 0.072	0.233 ± 0.041	0.322 ± 0.048	3.515 ± 0.499
24	1.656 ± 0.005	0.375 ± 0.001	0.526 ± 0.002	0.189 ± 0.002	0.228 ± 0.002	2.975 ± 0.007
25	0.837 ± 0.020	0.122 ± 0.002	0.443 ± 0.004	0.292 ± 0.001	0.332 ± 0.010	2.025 ± 0.025
26	0.762 ± 0.004	0.218 ± 0.001	0.326 ± 0.001	0.180 ± 0.012	0.207 ± 0.014	1.693 ± 0.019
27	0.695 ± 0.011	0.237 ± 0.004	0.278 ± 0.004	0.151 ± 0.008	0.182 ± 0.006	1.543 ± 0.032
28	0.957 ± 0.073	0.186 ± 0.016	0.182 ± 0.014	0.324 ± 0.045	0.387 ± 0.052	2.037 ± 0.200

Data from Table 4 were used to calculate the effect of each independent variable (growth parameter) on the accumulation of each carotenoid (dependent variable), using a statistical significance lower than 10% to identify the most significant independent factors (Table 5). Fx production by *P. gyrans* was promoted by decreasing light intensity (*p* < 0.001) and increasing NaNO_3_ (*p* = 0.006), which means that these variables had a negative and positive effect on this carotenoid, respectively. These variables also had a similar impact on Ddx content, together with salinity, whose lower values increased the accumulation of Ddx (*p* = 0.031).

**Table 5:** Calculated effects for each carotenoid of *P. gyrans* from the Plackett–Burman experimental design.

	Fucoxanthin (mg g^−1^)		Diatoxanthin (mg g^−1^)		Diadinoxanthin (mg g^−1^)		β-carotene (mg g^−1^)		α-carotene (mg g^−1^)		Total Carotenoids (mg g^−1^)	
	Effect	*p*-value	Effect	*p*-value	Effect	*p*-value	Effect	*p*-value	Effect	*p*-value	Effect	*p*-value
Mean	1.402	**0.000**	0.462	**0.000**	0.391	**0.000**	0.287	**0.000**	0.299	**0.000**	2.840	**0.000**
Curvature	− 1.177	**0.052**	− 0.543	**0.020**	− 0.167	0.365	− 0.101	0.291	− 0.043	0.784	− 2.032	**0.023**
Inoculum size	0.020	0.923	0.170	**0.043**	− 0.016	0.816	0.044	0.226	0.008	0.894	0.226	0.441
Salinity	0.002	0.993	− 0.250	**0.007**	− 0.169	**0.031**	− 0.033	0.352	− 0.059	0.335	− 0.509	**0.103**
Light intensity	− 1.467	**0.000**	− 0.496	**0.000**	− 0.347	**0.001**	0.060	0.113	0.032	0.598	− 2.219	**0.000**
Air flow	0.155	0.457	0.094	0.227	0.060	0.387	− 0.033	0.357	− 0.046	0.450	0.230	0.433
NaNO_3_	0.703	**0.006**	0.218	**0.015**	0.195	**0.017**	0.079	**0.046**	0.101	0.114	1.295	**0.001**
NaH_2_PO_4_ H_2_O	− 0.128	0.534	− 0.189	**0.028**	− 0.045	0.511	− 0.029	0.410	− 0.037	0.538	− 0.429	0.160
Na_2_H_2_EDTA 2H_2_O	− 0.200	0.341	− 0.002	0.977	− 0.046	0.508	0.006	0.871	− 0.002	0.974	− 0.244	0.406
H_3_BO_3_	− 0.182	0.385	0.051	0.501	− 0.041	0.550	0.034	0.341	0.068	0.267	− 0.070	0.808
FeCl_3_ 6H_2_O	0.291	0.177	0.132	0.102	0.121	0.101	0.072	**0.062**	0.063	0.304	0.680	**0.038**
MnCl_2_ 4H_2_O	− 0.041	0.842	0.017	0.816	− 0.005	0.945	− 0.001	0.977	− 0.026	0.658	− 0.056	0.846
NaHCO_3_	0.175	0.402	0.269	**0.005**	0.039	0.569	− 0.005	0.879	− 0.032	0.590	0.446	0.146
ZnCl_2_	0.167	0.422	0.107	0.175	0.008	0.908	− 0.009	0.804	− 0.015	0.800	0.258	0.381
CoCl_2_ 6H_2_O	0.251	0.239	0.182	**0.033**	0.091	0.202	− 0.017	0.629	0.001	0.992	0.508	**0.104**
(NH_4_)6Mo_7_O_24_ 4H_2_O	− 0.019	0.927	0.075	0.330	0.035	0.607	− 0.050	0.173	− 0.089	0.156	− 0.048	0.868
CuSO_4_ 5H_2_O	− 0.011	0.956	0.023	0.753	− 0.004	0.949	− 0.021	0.558	− 0.012	0.845	− 0.025	0.931
Thiamin	0.302	0.163	0.031	0.674	0.041	0.553	0.039	0.275	0.033	0.586	0.446	0.146
Cyanocobalamin	0.078	0.703	0.011	0.883	− 0.029	0.675	− 0.029	0.410	− 0.043	0.478	− 0.012	0.967

Bold numbers represent the abiotic factors with *p*-values considered statistically significant (*p* < 0.10).

Results showed that βCar was positively correlated with higher supplementation of NaNO_3_ (*p* = 0.046) and FeCl_3_.6H_2_O (*p* = 0.062), whereas for αCar none of the independent variables was statistically significant within the tested ranges (*p* > 0.10). Dtx was identified as the carotenoid with the highest number of significant variables (7). Concerning the sum of all carotenoids of *P. gyrans*, three abiotic factors with statistical importance were identified: light intensity (*p* < 0.001), NaNO_3_ (*p* = 0.001), and FeCl_3_.6H_2_O (*p* = 0.038). As already mentioned for individual carotenoids, nitrogen and copper concentrations in the medium were positively correlated with total carotenoids content, in contrast to the light intensity. In order to avoid excluding any important variable, the nutrient CoCl_2_.6H_2_O and the salinity level of the medium were also considered significant variables, given the closeness of their *p*-values (*p* = 0.103–0.104) to the statistical significance threshold adopted. Among the dependent variables assessed, only Fx, Dtx, and TCar presented a significant curvature (*p* < 0.10), meaning that the optimal value for these carotenoids was reached within the range of abiotic factors tested. Overall, the abiotic factors here identified with a marked effect on the carotenoid composition of *P. gyrans* are in accordance with described in literature, and their effects are discussed below.

#### Light intensity

Light plays a crucial role in photosynthetic organisms, providing the energy needed for the carbon fixation that will be metabolized for further biocompounds synthesis and cell growth. Similarly to the results here reported for *P. gyrans*, increasing light intensity in nitrogen-stressed cells of *D. lutheri* contributed to a sharp decrease (> 50%) in total carotenoids content^32^. These authors argued that, at higher light intensities, the N-starvation condition triggered the accumulation of Ddx and Dtx, mainly at the expense of βCar. Similarly, *P. gyrans* grown under three different illumination levels achieved the lowest fucoxanthin/chlorophyll *a* ratio and greater growth rates with increasing light intensity. Higher light intensity led to an overall decrease in pigment content, both in photosynthetic and photoprotective pigments^33^. As reported for the haptophyte *Emiliania huxleyi*, the photoacclimation process has as the main goal to optimize photosynthesis efficiency by changing the composition of the LHC. At low light intensity, this is reached by increasing the amounts of photosynthetic pigments and proteins LHCF^34^. LHC of *P. gyrans* is composed by a Fx-Chl a/c-protein complex^31^, which explains the negative effect of the light intensity over the pigments composition here reported.

#### Nitrogen

Regarding the NaNO_3_, a comparable trend was described for the Fx content of *Pavlova* OPMS 30,543, whose value increased in culture medium supplemented with higher levels of nitrate^35^. Such trend was also stated in other microalgae genus like *Phaeodactylum*^36^ and *Odontella*^37^. Likewise, the total carotenoids content of *P. pinguis* had a substantial increase (58 times) when the nitrogen supplementation changed from 140 to 1752 μmol L^−1^ NaNO_3_^38^. Longworth et al.^39^ verified that nitrogen-stressed *P. tricornutum* cells showed a significant reduction in the photosynthetic pathway due to the lower abundance of enzymes responsible for carbon fixation, as well less presence of proteins and pigments in the photosynthetic apparatus. The reason for that relies on its chemical composition, which possesses a high content of nitrogen. Therefore, to maximize the nitrogen availability for protein synthesis indispensable for cell subsistence, stressed cells down-regulate the photosynthetic pathway, causing a decrease in their pigments^39^.

#### Salinity

Under salinity stress, microalgae tend to increase the intracellular composition of the signaling molecules, such as calcium and reactive oxygen species (ROS)^40^. By reacting with macromolecules (*e.g.*, DNA, proteins), ROS can severely impair the cell metabolism or even lead to death. In order to counteract ROS action, cell activates antioxidant defense mechanisms, which may involve increased production of antioxidant compounds^40,41^. Overall, salinity stress has been especially highlighted as a key factor responsible for causing the accumulation of carotenoids in several microalgae from the genus *Dunaliella*^42^, *Chlorella*^43^, *Desmodemus*^44^, and *Haematococcus*^40^. For instance, *Dunaliella tertiolecta* grown under extreme salinities (3 M) was shown to have a marked increase—at cellular basis—of βCar, although the negative impact in the microalgae growth performance^42^. *Desmodesmus* sp. also presented a total carotenoid content 2.7-fold higher when subjected to salt stress conditions^44^.

However, the TCar of *P. gyrans* (Table 5) had a negative impact promoted by salinity. Despite the different salinity range tested, the Fx content of marine species *Tisochrysis lutea* and *P. tricornutum* also presented a significant drop when grown with increasing salinities beyond the optimal level (35–45 ‰)^45^. The authors also discussed that, although the suboptimal salinity levels tested led to stressful conditions for the photosynthetic apparatus of the cells, the impact on photosynthetic pigment production (*e.g.*, fucoxanthin) remains unclear. This particular evidence points out the species-dependent behavior of each microalga for the same abiotic factor.

#### Iron

Iron is an important micronutrient for several cellular processes, such as DNA synthesis, respiration, and photosynthesis. It is a crucial cofactor for enzymatically mediated processes, which allow the physiological and chemical balance, as well as a key nutrient for chlorophyll synthesis and chloroplasts stability^46^, and the antioxidant mechanism against ROS^47^. In fact, Fe limitation has been described as a limiting nutrient in oceanic waters, even those with higher concentrations of macronutrients (i.e. N and P)^48^. In this work, the increasing concentrations of iron within the range tested showed a positive effect on carotenoids accumulation of *P. gyrans*. Fe is a key component in the nitrogen assimilation, a nutrient itself that develops a crucial role in the photosynthetic pathways, as previously explained in the “Nitrogen” section. The overall increase of carotenoids at higher Fe supplementation may be explained by its high concentration in the chloroplasts and, especially, in the photosystem I (PSI)—12 Fe per PSI^47^. PSI and PSII are pigment-protein complexes which may contain different pigments, namely chlorophyll a, c_1_ + c_2_, beta-carotene, and xanthophylls, being the Fe availability strictly connected to the pigment modulation of the PS^49^. The deficient supply of iron in *Chlamydomonas reinhardtii* induced the remodeling of LHCI, by processing and up- or down regulation of its pigment-binding proteins, with a concomitant decrease of excitation energy efficiency to the PSI^47^. Kosakowska et al.^48^ reported a similar trend in *P. tricornutum*, whose cells were grown in a range of 0.001 to 10 µmol L^−1^ Fe^3+^, with higher concentrations promoting the highest contents of chlorophyll *a*, *c*_*1*_ + *c*_*2*_, Fx, Ddx, and βcarotene. The authors also noticed that the iron-deficient cells presented a marked decrease of its βCar, a conclusion that is in line with *P. gyrans* considering the relevance (*p* = 0.062) achieved for FeCl_3_.6H_2_O (Table 5)**.**

#### Cobalt

Cobalt is an important micronutrient for microalgae, mainly due to its role as structural molecule involved in the synthesis of the important cofactor cobalamin, which in turn influences the enzymatic processes responsible for nitrogen fixation^50^. However, when supplied at higher concentrations, this heavy metal can be harmful for microalgae as consequence of the increasing oxidative stress^51^, a phenomenon always described as species-dependent. In the present work, cobalt had a positive impact on TCar, Dtx, and αCar content of *P. gyrans.* Indeed, the increasing accumulation of protective carotenoids (*e.g.*, Dtx) under higher cobalt concentrations might be seen as the response of *P. gyrans* against the likely production of ROS during electron acceptor–donor interactions of cobalt^52^. The supplementation of cobalt at low levels proved to be successful for microalgae growth and carotenogenesis in *Spirulina platensis*^53^*, Monoraphidium minutum*, and *Nitzchia perminuta*^54^*.* Although the decreasing content in both classes of pigments, the authors also highlighted the superior stability of carotenoids at higher cobalt levels, in contrast to chlorophyll^54^*.*

For practical reasons, only three responses (those allowing to maximise Fx, βCar, and TCar) were considered in the validation assays. The aim was to confirm the potential of *P. gyrans* as a carotenoid producer, in particular of the commercially important and demanded βCar and, mostly, Fx. Considering the interconnected and dependent production of Dtx and Ddx (xanthophyll cycle), the individual validation of these carotenoids was passed over in favor of TCar.

### Validation tests

#### Growth analysis

**Figure 1:**
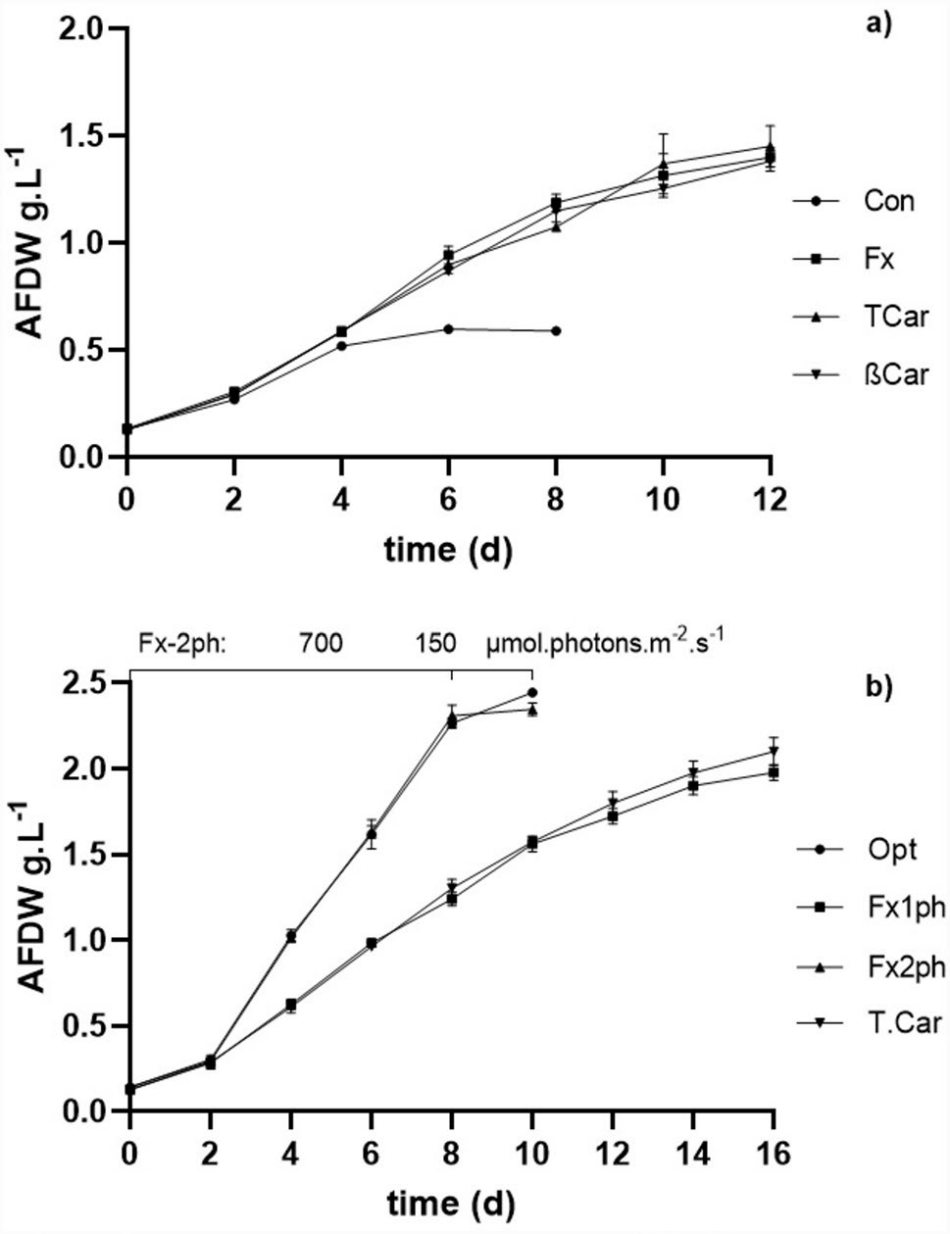
Growth profile of *P. gyrans* cultivated with the modified medium for the validation experiments V1 (**a**): Walne's medium (Con) and the maximized conditions for accumulation of fucoxanthin (Fx), β-carotene (βCar), and the sum of all carotenoids analyzed (TCar). In V2 (**b)** was assessed the optimized growth conditions for *P. gyrans’* biomass production (Opt) and the maximized growth conditions for TCar and fucoxanthin: 150 µmol photons m^−2^ s^−1^ during the entire growth (Fx1ph) or using 700 µmol photons m^−2^ s^−1^ for the first 8 days and 150 µmol photons m^−2^ s^−1^ for the last 2 days (Fx2ph). The experiments were performed in triplicate and the error bars represent the mean values and standard deviation.

The culture conditions applied in the validation assays are presented in Table 3. The growth performance of *P. gyrans* under the validation conditions of set V1 is presented in Fig. 1. Despite the decreasing concentration in ten nutrients, *P. gyrans* grown in the assay Fx, βCar, and TCar had longer growth and higher *X*_*max*_ values when compared to the control medium (Con) (see Supplementary Table S1). Among the same seventeen growth parameters, nitrogen supplementation, along with light intensity and copper, were identified in a previous work of our research group as the most important factors in biomass production of *P. gyrans*^25^. As the Fx, βCar, and TCar maximization assays share the same levels of NaNO_3_ (7.5 times higher than Con) and copper (2 times lower than Con), the ≈twofold increase in final *X*_*max*_ and *P*_*x*_ may be explained by the variation in these growth conditions.

Regarding the validation set V2, it was verified that *P. gyrans* grown in the assay Opt.V2 and Fx2ph.V2 showed significantly higher *X*_*max*_ and *P*_*x*_ compared to the experiments Fx1ph.V2 and TCar.V2 (see Supplementary Table S1). The improved biomass production in Opt.V2 and Fx2ph.V2 may be related to the higher illumination, which was used during the whole growth and until the end of the exponential growth phase, respectively. In fact, light intensity stood out as the most significant variable in our previous work aimed at optimizing biomass production of *P. gyrans*^25^, in which its optimal value was defined as 700 µmol photons m^−2^ s^−1^, the same used in Opt.V2 and Fx2ph.V2. The increase of light intensity from 150 to 700 µmol photons m^−2^ s^−1^ was responsible for shortening by 6 days the growth of *P. gyrans*, which almost doubled its volumetric productivity (see Supplementary Table S1). Several works have described the increase in the growth performance with light intensity^33,55^.

#### Carotenoids composition

**Figure 2:**
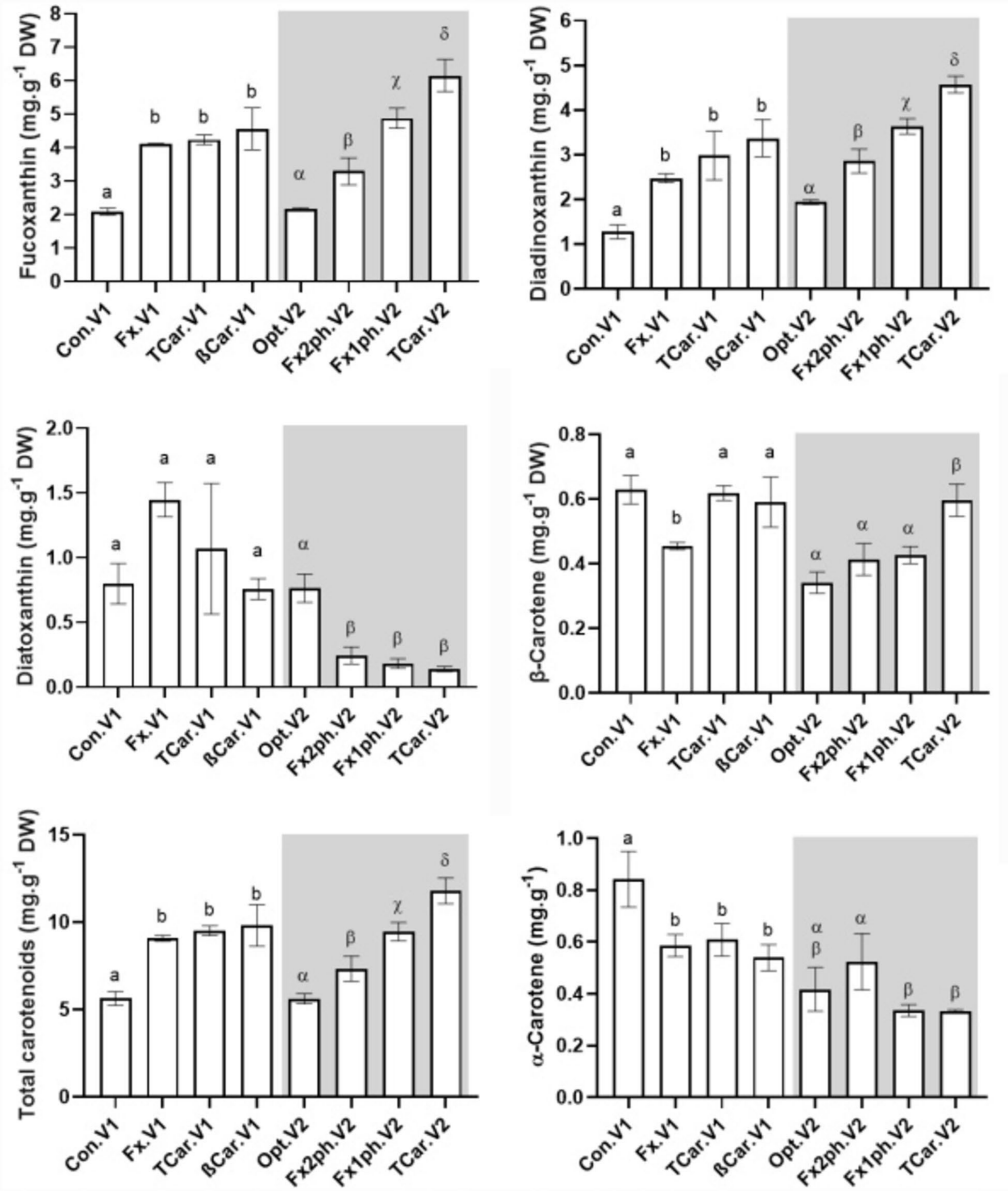
Carotenoids composition, mg g^−1^, of *P. gyrans* cultured in validation experiments (V1 and V2). In V1, was evaluated the Walne's medium (Con) and the maximized conditions for accumulation of fucoxanthin (Fx), β-carotene (βCar), and the sum of all carotenoids analyzed (TCar). In V2 (**b**) was assessed the optimized growth conditions for *P. gyrans’* biomass production (Opt) and the maximized growth conditions for TCar and fucoxanthin: 150 µmol photons m^−2^ s^−1^ during the entire growth (Fx1ph) or using 700 µmol photons m^−2^ s^−1^ for the first 8 days and 150 µmol photons m^−2^ s^−1^ fot the last 2 days (Fx2ph). The assays were performed in triplicate, with the bars representing the mean values and the standard deviation. Bars over the grey background represent the values produced by the validation test 2 (V2). Means with different letters within each data set (V1 or V2) are significantly different (*p* < 0.05).

Carotenoids composition of *P. gyrans* produced in the validation experiments is described in Fig. 2.

Although the growth conditions tested were primarily aimed at maximizing Fx, βCar, and TCar content, the results obtained for Dtx, Ddx, and αCar were also presented in all experiments. Regarding the Fx content, in validation set V1 it was seen that Con.V1 produced 2.010 mg g^−1^ DW, nearly half of the concentration achieved in the remaining experiments (Fx.V1, TCar.V1, and βCar.V1). The Fx values between Fx.V1, TCar.V1, and βCar.V1 were similar (*p* > 0.05), which can be justified by using the same level of the most significant variables for this carotenoid (light intensity and NaNO_3_). Similarly, in validation set V2, the control experiment (Opt) presented the lowest level of Fx, with 2.154 mg g^−1^ DW (*p* < 0.05). The two approaches carried out to improve Fx accumulation in *P. gyrans* were succeeded, with Fx2ph.V2 and Fx1ph.V2 reaching 3.294 and 4.879 mg g^−1^ DW, respectively. Although the Fx content produced was lower than that achieved in the assays performed strictly at low light intensity, the approach tested on Fx2ph.V2 promoted a 1.5-fold increase in Fx concentration compared to Opt, without compromising biomass production (Fig. 1 and Supplementary Table S1). In opposition to V1, *P. gyrans* grown under TCar.V2 conditions promoted the highest Fx content (*p* < 0.05), whose value reached 6.153 mg g^−1^ DW. Such increase might be explained by a richer nutrient composition in V2, among which the notable increase in the NaNO_3_ level (1500 vs 750 mg L^−1^) stands out.

Ddx presented a similar trend to that of Fx. In V1 set, all the conditions tested yielded higher Ddx contents than the control conditions (*p* < 0.05), which values ranged from 1.273 to 3.368 mg g^−1^ DW. In the V2 set, Opt showed, once again, the lowest content of Ddx (1.938 mg g^−1^ DW), with a significant increase in the accumulation of this carotenoid occurring as light intensity decreased, reaching its maximum in TCar.V2 (4.573 mg g^−1^ DW). With respect to Dtx, the conditions tested in V1 showed no significant differences, with the highest content produced by *P. gyrans* found in Fx.V1 (1.448 mg g^−1^ DW) and the lowest achieved in βCar (0.758 mg g^−1^ DW). Dtx profile had the opposite trend of the Ddx in V2 set. Dtx values ranged from 0.139 to 0.763 mg g^−1^ DW, with the highest value being reached under high illumination (Opt.V2) and considered significantly higher than the remaining assays (*p* < 0.05). The relationship between these xantophylls is a well-known process (diadinoxanthin cycle) widely described in several haptophytes and diatoms, that is triggered against the oxidative stress promoted by high light conditions. Through enzyme-mediated processes, microalgae produce Dtx by de-epoxidation of Ddx at high irradiances; conversely, epoxidation of Dtx to Ddx occurs at low light intensities^52^. Due to its higher number of the conjugated double bonds^56^, Dtx proved increased performance in scavenging of free radicals and quenching of chlorophyll triple states, protecting and stabilizing the thylakoid membranes under high irradiances and temperatures^8,52^.

Among the carotenoids optimized, βCar had the lowest content. There were no significant differences between Con.V1, TCar.V1, and the theorical optimal conditions (βCar.V1) with their contents ranging between 0.455 and 0.629 mg g^−1^ DW. Fx.V1 produced the lowest value of βCar (*p* < 0.05). Considering that FeCl_3_.6H_2_O was identified in the PB design as a key factor for the production of βCar by *P. gyrans*, its concentration may explain the low content of this carotenoid in Fx.V1. As displayed in Table 3, this assay was the only one that showed limited iron concentrations, thus validating the importance of the statistical approach conducted in this work. This observation is also corroborated by the results of V2. Although *P. gyrans* was not tested with culture conditions to increase the accumulation of βCar, like the βCar.V1, the only assay with a significant increase in this carotenoid was TCar.V2 (0.596 mg g^−1^ DW), which was also the only assay with a higher iron supplementation (1.95 mg L^−1^). Regarding the αCar, its value ranged between 0.540–0.843 and 0.322–0.524 mg g^−1^ DW in the V1 and V2 experiments, respectively.

Considering that the most prominent carotenoids in *P. gyrans* were Fx and Ddx, these carotenoids strongly influenced the TCar content. In fact, TCar content among the assays matched the profile already described for Fx and Ddx. In the set V1, Con produced the lowest content (*p* < 0.05) of total carotenoids, 5.633 mg g^−1^ DW, whereas in Fx.V1, TCar.V1, and βCar.V1 the range was 9.070–9.820 mg g^−1^ DW. On the other hand, all the experiments of the set V2 presented significant differences among them (*p* < 0.05). Considering the carotenoids content, whose value ranged from 5.614 to 11.794 mg g^−1^ DW, the validation experiments can be ranked as TCar.V2 > Fx1ph.V2 > Fx2ph.V2 > Opt.V2. Indeed, the results reported here for TCar validate the findings produced with the statistical approach adopted. In both V1 and V2, the manipulation of the main abiotic factors according to PB (salinity, light intensity, NaNO_3_, FeCl_3_.6H_2_O, and CoCl_2_.6H_2_O) proved successful, allowing a 1.7- and 2.1-fold increase in the final composition of *P. gyrans*, respectively.

Taking into consideration the assay with the highest yield in carotenoids (TCar.V2), the statistical approach here adopted led to a modified medium with improved production of Fx (6.153 mg g^−1^ DW) and TCar (11.794 mg g^−1^ DW). *P. gyrans* grown under those conditions showed a higher content of carotenoids compared to *D. lutheri* (5–6 mg g^−1^ AFDW)^32^ and *P. pinguis* (4.32–2.91 mg g^−1^ DW) ^38,57^. On the other hand, the Fx content of *Pavlova* sp. OPMS 30,543, grown under optimized growth conditions, reached 12.88 mg g^−1^ DW at lab-scale, which rose to 20.86 mg g^−1^ DW when grown at outdoor conditions^35^. Beyond the Pavlovophyceae, other species are well-known producers of carotenoids, especially Fx. Depending on the cultivation features, the Fx content described for the haptophyte *Tisochrysis lutea* was 5.51–10.73 mg g^−1^ ^58^. *Phaeodactylum tricornutum*^36^ and *Odontella* aurita^37^ were also highlighted as highly productive species, whose Fx content achieved was 42.8, 18.47, 18.18 mg g^−1^, respectively.

#### Carotenoids productivity

**Figure 3:**
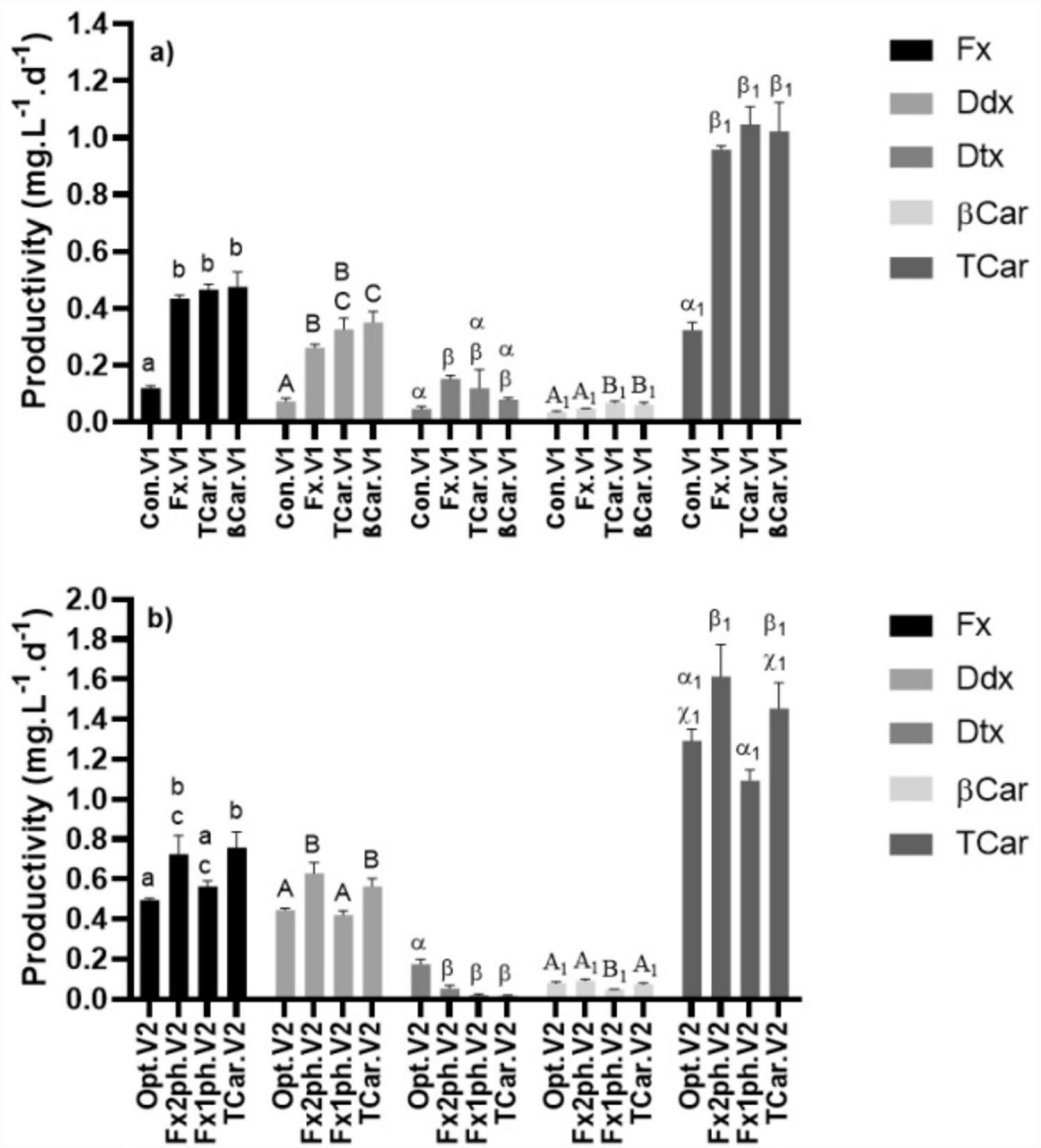
Volumetric productivities of the carotenoids (fucoxanthin, Fx; diadinoxanthin, Ddx; diatoxanthin, Dtx; β-carotene, βCar; total carotenoids, TCar), mg L^−1^ d^−1^, of *P. gyrans* grown in validation conditions V1 (**a**) and V2 (**b**). In V1, was evaluated the Walne's medium (Con) and the maximized conditions for accumulation of fucoxanthin (Fx), β-carotene (βCar), and the sum of all carotenoids analyzed (TCar). In V2 (**b**) was assessed the optimized growth conditions for *P. gyrans’* biomass production (Opt) and the maximized growth conditions for TCar and fucoxanthin: 150 µmol photons m^−2^ s^−1^ during the entire growth (Fx1ph) or using 700 µmol photons m^−2^ s^−1^ for the first 8 days and 150 µmol photons m^−2^ s^−1^ for the last 2 days (Fx2ph). Bars with different superscript letters are significantly different (*p* < 0.05).

The data from growth performance (Fig. 1) and the respective carotenoids content (Fig. 2) were used to calculate carotenoids productivity (mg L^−1^ d^−1^) of *P. gyrans* in the validation experiments (Fig. 3). In general, the values obtained for Con.V1 are in line with the previously described in “Carotenoids composition” section. The low biomass production, together with the low content of carotenoids—especially Fx, Ddx, and TCar—made the respective productivity values significantly lower than in the other assays (Fig. 3a). Furthermore, the different values of βCar productivity compared between Con.V1 and βCar.V1 should be pointed out. Although βCar.V1 showed a 4-day increase in growth duration, as well as lower βCar content, this assay achieved higher productivity than Con.V1. This improvement was due to the higher *X*_*max*_ achieved, which almost doubled in comparison with Con.V1 (see Supplementary Table S1). Thus, although βCar.V1 failed to increase βCar content (Fig. 2), these growth conditions proved advantageous for enhancing its productivity.

In the set V2, it was found that TCar.V2 and Fx2ph.V2 stood out as the most productive conditions for Fx (0.726–0.759 mg L^−1^ d^−1^), Ddx (0.564–0.629 mg L^−1^ d^−1^), and TCar (1.454–1.615 mg L^−1^ d^−1^). The management of light intensity (Fx2ph.V2) throughout *P. gyrans* growth resulted in important gains in productivity for Fx and TCar, ranking the Fx2ph.V2 as the second and first most productive assay, respectively. Thus, the reduction from 700 to 150 µmol photons m^−2^ s^−1^ in the last 2 days of growth can be seen as an interesting strategy for the maximization of *P. gyrans’* biomass production, along with higher productivity for the biologically important carotenoids.

Looking at the pigment fucoxanthin, its maximum productivity value achieved with *P. gyrans* (0.759 mg L^−1^ d^−1^) is within the range described for other species that were studied as a fucoxanthin source, such as *Chaetoceros muelleri* (0.072 mg L^−1^ d^−1^)^45^, *P. tricornutum* (0.041–2.3 mg L^−1^ d^−1^)^36,45^, but on the other hand, it falls short of the productivity described for *Tisochrysis lutea* (4.71 mg L^−1^ d^−1^)^58^. Although fucoxanthin productivity of *P. gyrans* is below some of the values recorded for *P. tricornutum* and *T. lutea,* it must regarded as a promising alternative source of fucoxanthin. In particular, *Pavlova* species have some interesting characteristics, namely: a high content of omega-3 fatty acids; the absence of a rigid cell wall, which enhances their digestibility; and a perfectly balanced ratio between essential and non-essential amino acids, which makes them a complete source of bioactives for further application at industrial scale and as a valuable new ingredient for human consumption.

## Conclusion

In this work, a multivariate approach was implemented to identify the significant growth parameters on carotenoids’ composition of *P. gyrans* (*p* < 0.10)*.* Fx content was mainly affected by the light intensity and NaNO_3_, whereas TCar content was influenced by light intensity, NaNO_3_, salinity, cobalt, and iron. The manipulation of the key abiotic factors proved successful due to the generalized increase in Fx and TCar. The highest Fx (6.153 mg g^−1^ DW) and TCar (11.794 mg g^−1^ DW) contents were achieved in the same experiment, in which the key variables were set to increase the total carotenoids content.

### Supplementary Information

Supplementary Information.

## Data Availability

Data is available upon reasonable request through the following email: jfilipemaciel@ceb.uminho.pt.
