# Supplementary material for: Identification and optimization of the key growth parameters involved in carotenoids production of the marine microalga Pavlova gyrans
Source: Sci Rep. 2024 Jul 26;14:17224. doi: 10.1038/s41598-024-66986-y (PMC11282313; doi:10.1038/s41598-024-66986-y)
Supplement: Supplementary file 1 — Supplementary Information. [file 41598_2024_66986_MOESM1_ESM.docx]

**Identification and optimization of the key growth parameters involved in carotenoids production of the marine microalga *Pavlova gyrans***

Filipe Maciel^1,2^, Paulo Berni^1,2^, Pedro Geada^1,2*^, José Teixeira^1,2^, Joana Silva^3^, António Vicente^1,2^

^1^CEB - Centre of Biological Engineering, University of Minho, Campus de Gualtar, Braga, Portugal,

^2^LABBELS –Associate Laboratory, Braga, Guimarães, Portugal

^3^ALLMICROALGAE, Natural Products S.A., Lisboa, Portugal

*Corresponding author:

E-mail: [pedrogeada@ceb.uminho.pt](mailto:pedrogeada@ceb.uminho.pt)

Postal address: Centre of Biological Engineering (CEB), University of Minho,

Campus de Gualtar, Braga, Portugal

Phone:+ 351 253 604 423

Supplementary Figure S1: Set-up of cultivation units used to grow *P. gyrans*


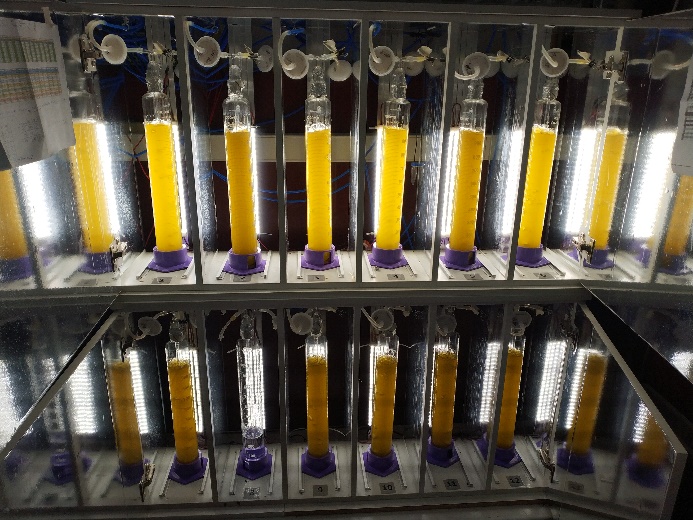


Supplementary Table S1: Average of three independent experiments, represented as mean ± standard deviation, of the maximum biomass produced, *X_max_*, and volumetric biomass productivity, *P_x_*, of *P. gyrans* grown in the validation experiments from the sets V1 and V2. In V1, Walne's medium (Con) was compared to the maximized conditions for accumulation of fucoxanthin (Fx), β-carotene (βCar), and the sum of all carotenoids analyzed (TCar). In V2 the maximized conditions for TCar and fucoxanthin - 150 µmol.photons.m^-2^.s^-1^ during the entire growth (Fx1ph) or using 700 µmol.photons.m^-2^.s^-1^ for the first 8 days and 150 µmol.photons.m^-2^.s^-1^ for the last 2 days (Fx2ph) - were compared with the optimized growth conditions for *P. gyrans’* biomass production (Opt). Values with different superscript letters are significantly different (*p* < 0.05)

|  | ***X_max_* (g AFDW.L^-1^)** | ***P_X_* (g AFDW.L^-1^.d^-1^)** |
| --- | --- | --- |
| **Con.V1** | 0.590 ± 0.010^a^ | 0.057 ± 0.001^a^ |
| **Fx.V1** | 1.400 ± 0.046^b^ | 0.106 ± 0.003^b^ |
| **βCar.V1** | 1.451 ± 0.096^b^ | 0.110 ± 0.008^b^ |
| **TCar.V1** | 1.380 ± 0.048^b^ | 0.104 ± 0.004^b^ |
| **Opt.V2** | 2.445 ± 0.010^A^ | 0.230 ± 0.002^A^ |
| **Fx1ph.V2** | 1.979 ± 0.048^B^ | 0.132 ± 0.004^B^ |
| **Fx2ph.V2** | 2.347 ± 0.037^A^ | 0.220 ± 0.004^A^ |
| **TCar.V2** | 2.102 ± 0.081^B^ | 0.123 ± 0.006^B^ |
